# Asymmetry in structure of the eggshell in *Osmylus fulvicephalus* (Neuroptera: Osmylidae): an exceptional case of breaking symmetry during neuropteran oogenesis

**DOI:** 10.1007/s00709-015-0860-z

**Published:** 2015-07-30

**Authors:** Arnold Garbiec, Janusz Kubrakiewicz, Marta Mazurkiewicz-Kania, Bożena Simiczyjew, Izabela Jędrzejowska

**Affiliations:** Department of Animal Developmental Biology, Institute of Experimental Biology, University of Wrocław, Sienkiewicza 21, 50-335 Wrocław, Poland

**Keywords:** Eggshell polarization, Dorso-ventral polarity, Follicular cell morphogenesis, Insect oogenesis, Insect ovaries, Neuroptera

## Abstract

Ovaries of neuropterans are of meroistic-polytrophic type. The ovarian tubes, the ovarioles, are divided into two major parts: a germarium, comprised of newly formed germ cell clusters; and a vitellarium, housing linearly arranged ovarian follicles. Each ovarian follicle consists of the germ cell cluster diversified into different number of nurse cells, and the oocyte enclosed by follicular epithelium. In *Osmylus fulvicephalus*, a representative of Neuroptera, during consecutive stages of oogenesis, the follicular cells undergo a multistep process of diversification which leads to the appearance of several follicular cell subpopulations i.e., the main-body follicular cells, the stretched cells, the anterior centripetal cells, and posterior centripetal cells. The anterior centripetal cells occupy the anterior pole of the oocyte and in advanced oogenesis due to hypertrophy that transform into anterior fold cells. Initially, the anterior fold cells form a symmetric fold, but in advanced oogenesis, quite different from other neuropterans studied so far, they undergo uneven hypertrophic growth which results in breaking symmetry of the anterior fold that becomes shifted to the ventral side of the oocyte. Since the anterior fold cells participate in the production of the specialized chorion structure, the micropyle, asymmetric structure of the anterior fold, is reflected both in its asymmetric position and in the asymmetric construction of the micropyle. As a consequence of breaking symmetry of the anterior fold, *Osmylus* eggshell gains dorso-ventral polarity, which is unusual for neuropterans.

## Introduction

Animal egg cells are one of the most remarkable voluminous cells in the organism because of the fact that they accumulate a great amount of organelles, macromolecules (proteins and RNAs), and reserve materials (yolk proteins, glycogen, and lipids) necessary for early developmental stages. In the vast majority of animals, the process of female gamete differentiation (oogenesis) occurs in ovaries and requires the involvement of various cells (somatic follicular cells and/or germline nurse cells) associated with the oocytes. Therefore, the different types of ovaries and modes of oogenesis have been distinguished. In solitary oogenesis, the oocyte grow without the support of the accompanying of cells, while in alimentary type of oogenesis in the oocytes growth, the soma and/or germ cells are involved.

The insect ovaries are composed of tubular units called ovarioles. Usually, the mature ovariole consists of three distinct parts: (1) the terminal filament, which anchors the ovariole to the fat body; (2) the germarium, where mitotic divisions of the germ cells take place; and (3) the vitellarium, occupied by a linear array of the ovarian units composed of germline and somatic cells called ovarian follicles. Two types of insect ovaries have been described: panoistic and meroistic (Büning 1994). In some panoistic and all meroistic ovaries, mitoses of the cystoblast are followed by incomplete cytokineses, thus, cystocytes remain connected by intercellular bridges forming germ cell clusters (Gottanka and Büning 1990; Rościszewska 1997; Büning 1994). In panoistic ovaries, all oogonia have potential to develop into oocytes. In meroistic ovaries within each cluster, one germ cell diversifies into an oocyte, whereas the remaining ones transform into nurse cells (trophocytes). In meroistic-telotrophic ovaries, nurse cells remain at the anterior part of the ovariole (tropharium), while oocytes in accompany of follicular cells develop in the vitellarium. The connection between oocytes and trophocytes is maintained by significantly elongated intercellular bridges called trophic cords. In meroistic-polytrophic ovaries, vitellarium houses linearly arranged ovarian follicles (= egg chambers) comprising germ cell clusters diversified into the oocyte and nurse cells covered by somatic follicular cells.

Both in panoistic and meroistic ovaries, germ cell differentiation and growth is accompanied by somatic epithelial cells termed the follicular cells. Those cells exhibit several peculiar features. The follicular cells are polarized cells, basally supported by the basement lamina and very frequently apically equipped with microvilli. Moreover, the follicular cells show also the ability to diversify into several distinct subpopulations that differ in morphology, behavior, function, and position in relation to the germline cells. Mechanisms that govern follicular cell diversification in insect ovaries are best known in a model organism, the fruit fly, *Drosophila melanogaster* (Margolis and Spradling 1995; Deng and Bownes 1998; Dobens and Raftery 2000; Tepass et al. 2001; Horne-Badovinac and Bilder 2005; Nystul and Spradling 2010). Comparative studies conducted on different insect groups reveal that the pattern of follicular cell diversification is group specific (Zawadzka et al. 1997; Kubrakiewicz et al. 2003; Mazurkiewicz and Kubrakiewicz 2005, 2008; Tworzydło et al. 2005; Żelazowska 2005; Ogorzałek 2007; Jaglarz et al. 2008, 2009, 2010; Garbiec and Kubrakiewicz 2012; Mazurkiewicz-Kania et al. 2012). It has been shown also that distinct subpopulations of follicular cells contribute to different processes that take place during oogenesis like e.g., vitellogenesis, establishing of the embryo polarity and eggshell formation (see e.g., Büning 1994).

An insect eggshell is an elaborate egg covering which comprises two major components: vitelline envelope and chorion (Kubrakiewicz et al. 2005). In insects, both the vitelline envelope and the chorion are synthesized by the follicular cells, and regional complexity of the eggshell reflects the diversification of the follicular cells. Comparative studies have shown that the insect eggshells exhibit a great variety of shape and architecture. One of the specialized regions of the chorion, produced by distinct subpopulations of the follicular cells, is a micropyle, a perforated region of the eggshell that enables sperm entry. The formation of the micropyle has been studied in a number of insect orders, e.g., Diptera (true flies) (Margaritis 1984; Zarani and Margaritis 1986, 1991a, 1994), Lepidoptera (moths and butterflies) (Yamauchi and Yoshitake 1984), Plecoptera (stoneflies) (Rościszewska 1995), Phthiraptera (true lice) (Zawadzka et al. 1997), Neuroptera (lacewings), Raphidioptera (snakeflies), and Megaloptera (alderflies, dobsonflies, and fishflies) (Kubrakiewicz et al. 2005). It has been shown that in some insect groups, e.g., Neuropterida (including Neuroptera, Megaloptera, Raphidioptera), the micropyle shows a complex structure serving not only for sperm entry but also for gas exchange (Kubrakiewicz et al. 2005).

Among insects with polytrophic ovaries, the course of oogenesis is the most extensively investigated in *Drosophila melanogaster*. Based on studies on *Drosophila*, it has been revealed that during oogenesis, the oocyte undergoes polarization and, in consequence, two orthogonal axes: antero-posterior and dorso-ventral, are established. The oocyte polarization is a hierarchical series of steps which comprise different symmetry-breaking events (Roth and Lynch 2009). The first step is selection of the oocyte, one cell within the cluster of the germ cells; second, posterior positioning of the oocyte within the egg chamber; third, a signal from the oocyte determines posterior cell fate within the follicular epithelium; and finally, asymmetric migration of the oocyte nucleus. Molecular analysis of axis formation in *Drosophila* has shown that essential for symmetry-breaking events is a TGFα-like ligand Gurken (Grk) which concentrates near the asymmetrically located oocyte nucleus. Of a great importance is a fact that this established polarization of the egg predeterminates future embryonic axes. In some insect groups, decoration of the eggshell with specialized chorion structures is closely related to the inner polarization of the oocyte. Such a correlation between the axial polarization of the egg, future embryo and the eggshell construction has been clearly demonstrated in *Drosophila melanogaster* (Queenan et al. 1997; Peri and Roth 2000).

Like in Diptera, the ovary of neuropteran insects is of meroistic-polytrophic type. The ovarioles house the ovarian follicles arranged linearly in subsequent stages of development. The pattern of follicular cell diversification in Neuroptera (lacewings) has been recently reported (Garbiec and Kubrakiewicz 2012). The aim of this study was to show that in a neuropteran insect *Osmylus fulvicephalus*, the course of follicular cell differentiation follows the basic program for neuropterans; however, in advanced stages of oogenesis, some modifications in follicular cells’ behavior occur. As a result, the eggshell displays not only antero-posterior but also dorso-ventral polarity, which is a unique feature among neuropteran insects. Both genesis and construction of asymmetries in the eggshell are discussed.

## Material and methods

### Insects

Specimens of *Osmylus fulvicephalus* used in this study were collected in SW Poland. For the study, the ovaries obtained from 30 adult females were used.

### Histological and ultrastructural analysis

For histological and ultrastructural observations, the ovaries from adult specimens were dissected and fixed at room temperature in 2.5 % glutaraldehyde in 0.1 M phosphate buffer (pH = 7.4). For convenience, the material was collected and kept in fixative for longer periods (usually for a few days) at +4 ^o^C. After fixation, the material was rinsed several times in phosphate buffer and postfixed for approximately 1 h in a mixture containing 1 % osmium tetroxide and 0.8 % potassium ferrocyanide. After dehydration in a graded acetone series, the ovaries were embedded in Epon 812 (Serva, Heidelberg, Germany). Semithin sections (0.6-μm thick) were stained with 1 % methylene blue in 1 % borax and examined with an Olympus BHS light microscope. Ultrathin sections (80-nm thick) were contrasted with uranyl acetate and lead citrate (Reynolds 1963) and examined in a Zeiss EM 900 at 80 kV.

For histological observation, the ovaries were also dissected and fixed in 4 % formaldehyde in phosphate-buffered saline (PBS: NaCl 137 mM, KCl 2.7 mM, Na_2_HPO_4_ 8 mM, KH_2_PO_4_ 1.5 mM). After a few rinses with PBS, the material was dehydrated in a graded series of ethanol and embedded in acrylic resin Histocryl (Sigma, H4396).

### Histochemical analysis

#### Whole-mounts

The ovaries were dissected and fixed in 4 % formaldehyde in phosphate-buffered saline (PBS) containing 0.1 % Triton X-100. After a few rinses with PBS, the material was first examined with the light microscope equipped with Nomarski optics, and then subjected to whole-mount fluorescent staining.

For detection of cell nuclei (DNA), the material was stained with 0.2 μg/ml DAPI (4′,6-diamidino-2-phenylindole dihydrochloride) (Sigma, D9542) for 20 min in darkness. For detection of microfilaments (F-actin), the ovaries were stained with 2 μg/ml rhodamine-conjugated phalloidin (Sigma, P1951) for 20 min in darkness. In both cases, after rinsing with buffer, the ovarioles were whole-mounted onto microscope slides and examined with either the Olympus BHS light microscope equipped with an epifluorescence device or with an Olympus FV1000 confocal microscope.

### Analysis of the eggshell surface with scanning electron microscope

For scanning electron microscope (SEM) studies, the eggs were removed from the oviducts and fixed in 2.5 % glutaraldehyde in 0.1 M phosphate buffer (pH = 7.4) and postfixed in 2 % osmium tetroxide as described above. After the dehydration in a series of ethanol or acetone, the eggs were dried by an immersion and subsequent rapid evaporation of hexamethyldisilazane (Serva). The eggs were coated with gold and examined in scanning electron microscopes: JSM 5410 at 25 kV or Stereoscan (British Cambridge Instruments) at 15 kV.

## Results

### Gross morphology of the ovariole

**Fig. 1:**
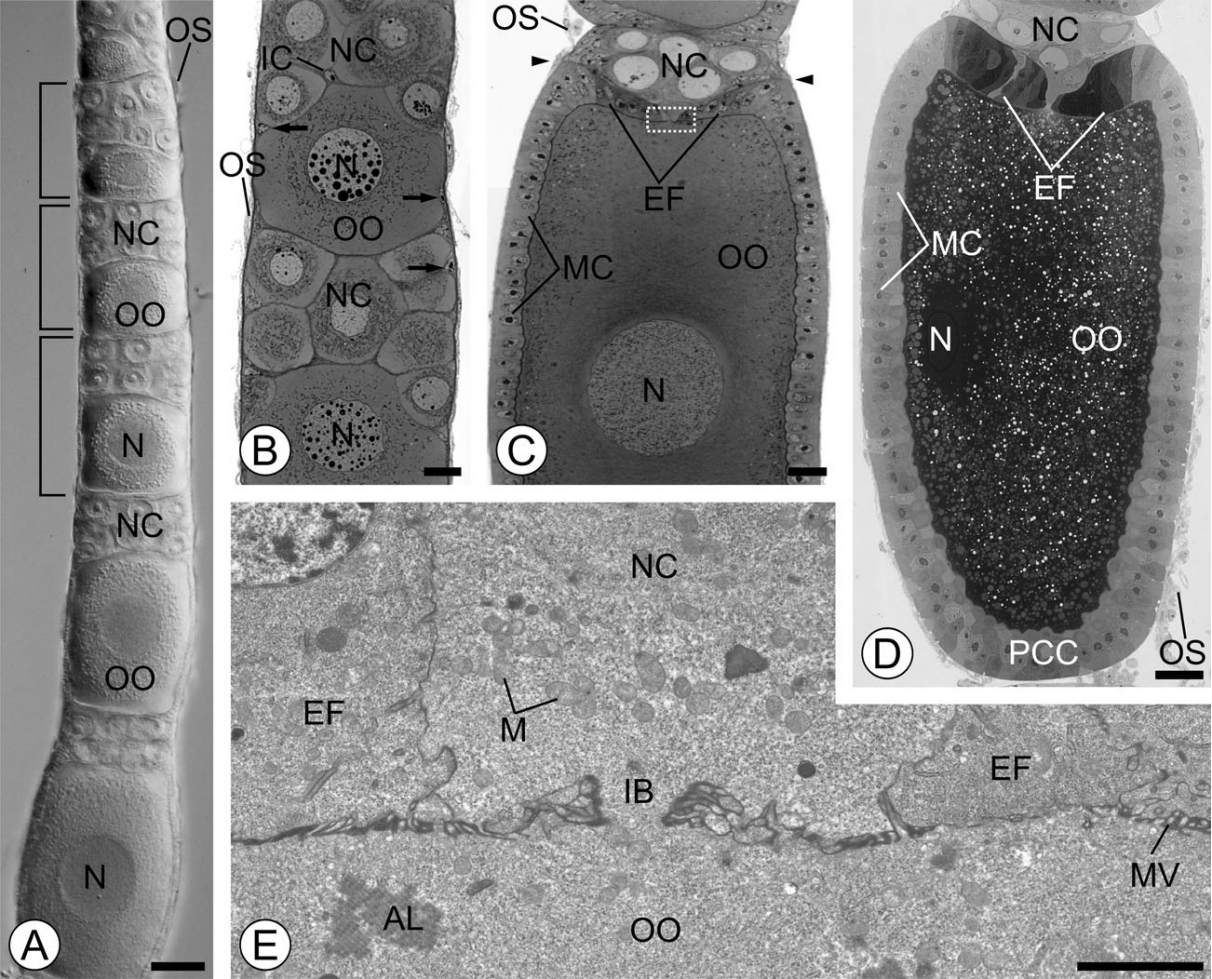
**a** The vitellarium of the ovariole in *O. fulvicephalus* is a linear array of egg chambers at progressively advanced stages of oogenesis along the antero-posterior axis. The anterior part of the vitellarium comprises previtellogenic egg chambers (denoted by *brackets*). In previtellogenic oocytes (*OO*), the nuclei (*N*) are located in the center of the cell. *NC* nurse cells, *OS* ovariolar sheath. Whole-mount preparation viewed with the Nomarski optics. *Scale bar* = 30 μm. **b** In previtellogenic egg chamber, both the oocyte (*OO*) and the nurse cells (*NC*) are laterally covered by small and flattened follicular cells (*arrows*). Oocyte nucleus (*N*) housing multiple nucleoli. *IC* interstitial cell, *OS* ovariolar sheath. **c** Egg chamber in the early vitellogenesis. Lateral sides of the oocyte (*OO*) are covered by cuboidal main-body follicular cells (*MC*). At the level of the nurse cell-oocyte boundary (marked by *arrowheads*), the follicular cells invade the nurse cell-oocyte interface and form a symmetric epithelial fold (*EF*) that progressively encloses the anterior pole of the oocyte (denoted by a *dotted rectangle* and shown at higher magnification (**e)**). Up to this stage of oogenesis, the oocyte nucleus (*N*) is still located centrally and filled with multiple nucleoli. Note that the egg chamber is ellipsoid. *OS* ovariolar sheath. **d** Mid-vitellogenesis. Oocyte nucleus (*N*) is shifted from a central to cortical (dorsal) position within the ooplasm. Follicular cells of the epithelial fold (*EF*) at the anterior pole of oocyte become conspicuously thicker. *MC* s main-body follicular cells, *PCC* posterior centripetal cells, *OS* ovariolar sheath. **b**–**d** Longitudinal semithin sections stained with methylene blue. *Scale bars* = 20 μm. **e** Early vitellogenesis. The intercellular bridge (*IB*) joins the nurse cell (*NC*) with the oocyte (*OO*). *AL* annulate lamellae, *EF* symmetrically located follicular cells of the epithelial fold, *M* mitochondria, *MV* oocyte microvilli. TEM. *Scale bar* = 2 μm

In *O. fulvicephalus*, the ovary is composed of ten elongated polytrophic ovarioles. In the ovarioles of adult females, the germarium houses already differentiated germline cells into well-recognizable nurse cells and oocytes. The vitellarium, which is a major part of the ovariole, consists of over a dozen, linearly arranged egg chambers at progressively more advanced stages of oogenesis. Each egg chamber comprises a cluster of sibling germ cells (an oocyte and nurse cells) (Fig. 1a, b) surrounded by a single-layered somatic follicular epithelium (Fig. 1b). Among the germ cells, somatic interstitial cells are intercalated (Figs. 1b and 3a, b). In *O. fulvicephalus*, consecutive egg chambers within the vitellarium are not separated from each other by any intercalating somatic structure (interfollicular stalk) (Fig. 1a, b). Each ovariole is externally covered by an ovariolar sheath (Fig. 1a–d). The ovariolar sheath is formed by epithelial cells and muscle fibers, both penetrated by tracheae. For more details on egg chamber morphogenesis in neuropteran ovaries, see Garbiec and Kubrakiewicz 2012.

### Differentiation of follicular cells from the early stages of previtellogenesis until advanced vitellogenesis

Newly assembled egg chambers are found in the anterior part of the vitellarium (previtellogenic growth zone) (Fig. 1a). Each egg chamber comprises an oocyte in the posterior position and the anterior grouping of nurse cells (Fig. 1a, b). The oocyte and the nurse cells progressively increase in size during subsequent stages of oogenesis, however, the relative proportions of their volume change conspicuously. From early previtellogenesis until mid-previtellogenesis, the oocyte is initially smaller, but then grows to equal the volume of the nurse cell compartment. During vitellogenesis, the volume of the nurse cell compartment decreases, whereas the oocyte becomes distinctly larger (Fig. 1d). During the period of rapid growth, the oocyte changes its shape. Initially roughly spherical, it becomes elongated along its antero-posterior axis (Fig. 1a).

**Fig. 2:**
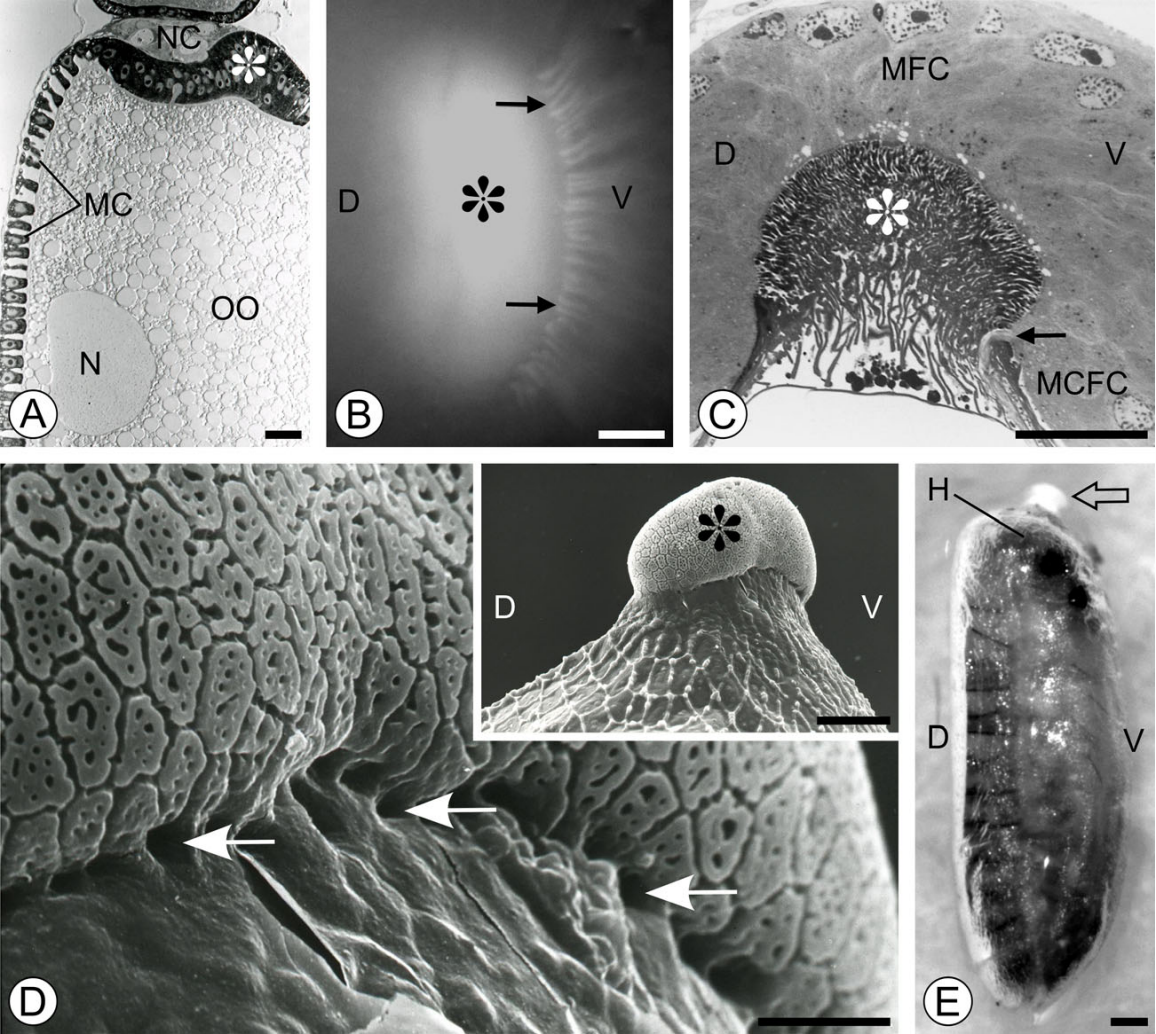
**a** Late vitellogenesis. Follicular cells forming the anterior fold (*asterisk*) are shifted off the antero-posterior axis of the egg chamber to a ventral position. *N* oocyte nucleus, *OO* oocyte, *MC* s main-body follicular cells, *NC* nurse cells. Semithin histocryl section stained with Azur B; *scale bar* = 40 μm. **b** Choriogenesis. At the cross section trough, the base of the forming micropyle and the long actin-rich cytoplasmic projections (*arrows*) of the micropyle canal-forming cells (MCFC) are visible at the ventral side of micropyle. The micropyle is denoted by *asterisk. D* and *V* mark the dorsal and ventral sides of the anterior epithelial fold, respectively. Whole-mount preparation stained with rhodamine-conjugated phalloidin; fluorescence microscope; *scale bars* = 50 μm. **c** Choriogenesis. Micropyle canal-forming cells (MCFC) are located at the ventral side of micropyle (asterisk), the long cytoplasmic projection of MCFC molds the micropylar canal. The micropyle is denoted by *asterisk. D* and *V* mark the dorsal and ventral sides of the anterior epithelial fold, respectively. Longitudinal semithin section stained with methylene blue; *scale bar* = 50 μm. (*D-insert*) Lateral view of the micropyle (*asterisk*) viewed in SEM. *D* dorsal side, *V* ventral side. *Scale bar* = 50 μm. **d** Details of the ventral side of the micropyle viewed in SEM. *Arrows* denote the openings of the micropylar canals. *Scale bar* = 10 μm. **e** Whole-mount preparation of the larva before its emergence from the egg shell. Outlines of the developing larva and its dorsal segmentation pattern can be seen through the semitranslucent egg shell. *Hollow arrow* marks the micropyle; *H* larval head; *D* and *V* denote dorsal and ventral side of the egg, respectively. *Scale bar* = 80 μm

**Fig. 3:**
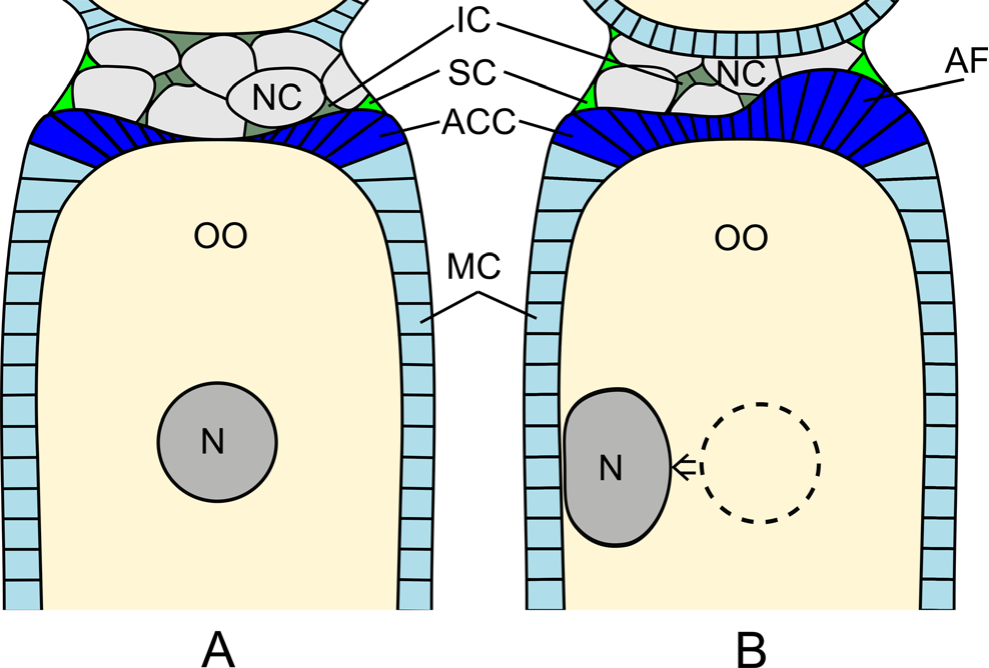
Schematic representation of the two steps of the anterior fold formation. **a** Early vitellogenesis. The oocyte (*OO*) is elongated and the oocyte nucleus (*N*) occupies central position. Main-body follicular cells (*MC*) cover lateral aspects of the oocyte, and anterior centripetal cells (*ACC*) encapsulate the anterior pole of the oocyte. The nurse cells (*NC*) are surrounded by (*SC*) and intermingled with interstitial cells (*IC*). **b** Mid-vitellogenesis. At the anterior pole of the oocyte hypertrophic centripetal cells form the asymmetric anterior fold (*AF*) at the ventral side of the oocyte. The germinal vesicle (*N*) is translocated from the cell center to the dorsal side

Germ cells in the egg chambers are surrounded by one-cell thick follicular epithelium throughout oogenesis. In the previtellogenic egg chambers, the follicular epithelium is uniformly squamous but not continuous, since it covers only the lateral aspects of the germ cells (Fig. 1b). During previtellogenesis and early vitellogenesis, the follicular cells proliferate, so their number considerably increases. These cells that surround the nurse cell compartment, in this report termed the stretched cells (SC), divide sporadically and remain flat. In contrast, those associated with the oocyte surface (main-body follicular cells (MCs)) multiply greatly, and so their number grows conspicuously. The increase in number of MCs is accompanied by a spectacular change in their shape. From initially flattened, they transform into clearly cuboidal (Fig. 1b–d). The follicular cells that are situated at the oocyte-nurse cell border, form centripetally directed protrusions, which penetrate between the oocyte and the nurse cells. These centripetally oriented cells gradually invade the nurse cell-oocyte interface dragging behind neighboring MCs (Fig. 1c). This invasion is radially symmetrical and so the apical tips of the centripetally oriented cells come to lie at the intercellular bridges that connect two nurse cells with the oocyte in the central part of its anterior pole (Fig. 1c, e). In consequence, at the beginning of vitellogenesis, the anterior pole of the oocyte is covered with the follicular epithelial layer. This covering, however, is incomplete because the oocyte is still connected with the nurse cells by the intercellular bridges. The anterior compartment of the egg chamber is covered only partially since its anteriormost pole is devoid of follicular cells. With the encapsulation of the oocyte by an almost continuous follicular cover, MCs become conspicuously thicker, and thus change from cuboidal to columnar (Fig. 1c). The thickness of the follicular epithelium is initially almost uniform over the whole surface of the oocyte (Fig. 1c). At mid-vitellogenesis, the MCs that surround the lateral aspects of the oocyte change their shape to more irregular and so are not tightly apposed to each other. At the same time, the centripetally oriented cells that overlay the anterior pole of the oocyte form a prominent, radially symmetric, anterior fold (Fig. 1d). Observations of semithin sections stained with methylene blue show that the cells of the anterior fold stain very intensely in comparison to neighboring MCs. The follicular cells of the posterior pole of the oocyte (posterior centripetal cells) morphologically resemble the MCs. At that stage, the connection between the nurse cells and the oocytes is maintained by means of still-functioning intercellular bridges (Fig. 1d). Towards more advanced stages of vitellogenesis, the cells of the anterior fold still grow and so become considerably taller. This growth, however, is uneven. The cells of the fold that are located ventrally (for dorso-ventral polarity of the egg chamber, see below) grow significantly, while those in the opposite, dorsal position, enlarge only slightly. As a result of uneven growth, the anterior fold is shifted off from the central position of the oocyte anterior pole, to an antero-ventral position (Fig. 2a and 3b).

In the final stages of oogenesis, the follicular cells participate in the formation of the eggshell that is deposited onto the surface of the oocyte. The MCs contribute to the deposition of a main-body eggshell, while the cells of the anterior fold form a micropylar apparatus (Fig. 2c). The micropyle-forming follicular cells of the anterior fold diversify into two distinct subpopulations: the micropyle-forming cells (MFCs) and the micropylar canal-forming cells (MCFCs). MFCs synthesize and secrete the material of the micropyle, while MCFCs located exclusively at the ventral side of the anterior fold are responsible for molding the micropylar canals, through which the sperms may gain access to the oocyte surface. MCFCs form elongated projections directed towards the surface of the oocyte anterior pole (Fig. 2b, c). Equipped with bunches of actin filaments (Fig. 2b), these projections penetrate into the forming micropyle.

Fully formed eggshell of *Osmylus* is differentiated into two distinct regions. The main body of the eggshell exhibits a characteristic polygonal pattern on its surface (Fig. 2d insert). The micropyle is a prominent, bean-shaped structure, elevated over the egg surface in the antero-ventral location (Fig. 2d, insert E). Its surface is porous and also shows a polygonal pattern (Fig. 2d). From the ventral side, the base of the micropyle is perforated (Fig. 2d). From these perforations, micropylar canals pass inward to the center of the micropyle where they bend towards the oocyte and open to the perioocytic space (Fig. 2c).

### Changes of shape and position of the oocyte nucleus during successive stages of oogenesis

During oogenesis in *Osmylus*, the oocyte nucleus (germinal vesicle) noticeably changes its size, shape, and position within the ooplasm. In early previtellogenic oocytes, it is relatively small, regularly spherical, and comprises numerous granules (Fig. 1b). Such granules have been already identified as the multiple nucleoli in germinal vesicles of neuropteran insects (see e.g., Kubrakiewicz, 2002). During previtellogenic growth, the oocyte nucleus gets visibly larger. The multiple nucleoli gradually grow in number, but instead their size is substantially reduced. Subsequently, in the early stages of vitellogenesis, the oocyte nucleus still increases in size, while the multiple nucleoli disappear almost completely (Fig. 1c). From the late stages of previtellogenesis, when the oocyte becomes elongated, and onward, the germinal vesicle can be found midway between the oocyte anterior and posterior poles (Fig. 1a, c). Roughly at the time, a prominent epithelial fold of follicular cells caps the anterior pole of the oocyte, the oocyte nucleus shifts its position from central to dorsal (Fig. 1d), but is still located halfway between the oocyte poles. In a new, subcortical location, the shape of the oocyte nucleus changes. Its surface facing the follicular epithelium conspicuously flattens, so the oocyte nucleus is not regularly spherical anymore (Fig. 2a).

### Axial polarity of the egg chamber and the eggshell in *O. fulvicephalus*

It is crucially important for the analysis of the follicular epithelium differentiation that the polarity of the egg chamber and the eggshell should be clearly defined. As it was indicated above, the axial polarity of the egg chamber is exemplified by asymmetric location of the anterior epithelial fold and the ultimate position of the oocyte nucleus. Whole-mount analysis of the position of the larva within the eggshell (Fig. 2e) shows that the dorsal side of the embryo develops from this part of the oocyte which contains the germinal vesicle. This analysis has been relatively simple since the eggshell in *Osmylus* is almost translucent, while the larvae do not change their position within the eggshell until eclosion. Thus, the “polarity markers”, such as the position of the larval head and location of the eyes and antennae, as well as the prominent segmental organization of the dorsal part of the larval cuticle, can be clearly viewed. The eggshell in *Osmylus* exhibits distinct and well-expressed antero-posterior and dorso-ventral axes. Axial polarity of the eggshell is best manifested by the antero-ventral position of the micropyle and its dorso-ventral asymmetry marked by the position of the micropylar canals (Fig. 2d, e).

## Discussion

### Peculiar arrangement of follicular cells and eggshell structure in *O. fulvicephalus*

Insect eggs and developing embryos are covered with envelopes that form an egg capsule (= egg shell). The egg shell protects against harmful environmental factors (mechanical damage, drying, bacterial infections), and on the other hand, enables gas exchange and the sperm entry. Usually, the egg shell is built of two layers: an inner vitelline membrane and an outer chorion. In most studied insects, the vitelline membrane is homogeneous and does not show any local modifications, while the chorion usually consists of several morphologically distinct sublayers and exhibits regional specialization, e.g., is equipped with structures that serve to attach the eggs to the substratum (attachment structures), respiratory apparatus (appendages), aeropylar openings (for gas exchange), an operculum which facilitates larval escape or a micropylar apparatus which allows sperm entry (for more details, see Büning, 1994).

In neuropterans, the only specialized and prominent region of the eggshell surface is the aeromicropyle which combines the functions of sperm entrance and gas exchange (Kubrakiewicz et al. 2005). In most lacewings, the aeromicropyle is formed at the anterior pole of the egg, although, in Myrmeleontidae and Ascalaphidae, the eggshells are equipped with two micropyles, located both at the anterior and posterior poles (Henry 1972; Kubrakiewicz et al. 2005). In this paper, we show that the *Osmylus* eggshell, like most lacewings, owns a single micropyle (aeromicropyle), however, dissimilar to other neuropterans, the micropyle is asymmetric both in structure and position. Since the chorion architecture reflects the pattern of follicular cell diversification, we followed subsequent stages of the egg chamber development in *O. fulvicephalus* to find out what modifications occur in the course of follicular cell diversification.

It has been previously reported that in neuropterans, follicular cell diversification is a multistep process (Garbiec and Kubrakiewicz 2012). During initial steps of the egg chamber development, two follicular cell subpopulations arise: the main-body follicular cells (MCs), covering lateral aspects of the oocyte; and the stretched cells, enclosing lateral aspects of the nurse cell compartment. With the progress of oogenesis, some MCs give rise to anterior and posterior centripetal cells which significantly elongate to encompass the anterior and posterior poles of the oocyte. As vitellogenesis proceeds, the anterior centripetal cells become hypertrophic and form a prominent anterior fold at the anterior pole of the oocyte. Eventually, the follicular cells of the anterior fold diversify into two distinct subpopulations that contribute to the micropyle formation (Kubrakiewicz et al. 2005; Garbiec and Kubrakiewicz, 2012). It has been previously demonstrated that in neuropteran insects, e.g., *Euroleon nostras* and *Oliarces clara*, micropyle canal-forming cells (MCFCs) are radially arranged, and thus the micropyle exhibits inner radial symmetry (Kubrakiewicz et al. 2005). Micropylar canals radiating from a center of the micropyle were described also in representatives of Ascalaphidae, such as *Ululodes mexicana* and *Ascaloptynx furciger* (Henry 1972). In this paper, we show that in *Osmylus*, the follicular cells share the general program of diversification with other studied lacewings. The similarities are manifested by occurrence of the same subpopulations of follicular cells. However, in *Osmylus*, the spatial arrangement of some follicular cell subpopulation is markedly dissimilar. Since advanced oogenesis the follicular cells of the anterior pole grow significantly, but only some of them, assembled at one side of the fold, become highly hypertrophic which leads to symmetry breaking of the anterior fold. As a consequence of uneven growth of follicular cells, the micropyle develops at the ventral side of the anterior pole and the eggshell gains DV asymmetry. Moreover, dissimilar to most lacewings, in *Osmylus*, all the MCFCs differentiate solely from the follicular cells gathered at the ventral side of the asymmetric fold, and so the openings of the micropyle are also ventrally located. Thus, uneven distribution of the micropylar canal-forming cells results in the asymmetric structure that is a characteristic of the *Osmylus* micropyle.

Among insects with polytrophic ovaries, the best studied with respect to the egg chamber development is the fruit fly *Drosophila melanogaster*. It should be emphasized, however, that *Drosophila* is a highly evolved fly with the most complex pattern of follicular cell diversification, and so the *Drosophila* system is not always suitable to be directly applied to other non-dipteran insects. One of the crucial differences concerns the fact that in *Drosophila*, the micropyle is produced by entirely different follicular cell subpopulations, namely, border and polar cells (Zarani and Margaritis 1986, 1991b). These cells diversify at the anterior pole of the egg chamber and display the ability for an invasive migration between the nurse cells to the anterior pole of the oocyte (Grammont and Irvine 2001; Montell 2003). Conversely, in Neuroptera, MFCs and MCFCs are recruited from centripetal cells. Engagement in micropyle formation of centripetally migrating follicular cells has been reported also in other insect groups, e.g., Lepidoptera (Telfer 2009; Mazurkiewicz-Kania personal communication).

Our knowledge of molecular mechanisms that govern the follicular cell differentiation in non-dipteran insects is very limited. Therefore, at this time, it is unknown as to what factors drive hypertrophic growth of the anterior fold cells found in most neuropterans and what mechanism is responsible for uneven growth of the anterior fold cells in *Osmylus*. To address the above-mentioned questions, further studies are needed.

### DV polarity of *Osmylus* eggshell does not correspond to DV polarity of the egg and future embryo

During oogenesis in *Osmylus*, the oocytes become polarized. As in other insects with polytrophic ovaries, the posterior position of the oocyte within the germ cell cluster concomitantly marks the posterior pole of the egg chamber and the antero-posterior (AP) axis of the oocyte, while asymmetric (dorsal) position of the germinal vesicle (GV) results in dorso-ventral (DV) polarity of the oocyte (van Eeden and Johnston 1999; Roth 2003). What is more, observations of transparent egg coverings in *Osmylus* allowed us to reveal that dorsal parts of the embryo body develop at the dorsal (GV housing) side of the oocyte, so DV polarity of the egg is consistent with DV polarity of the embryo. On the other hand, we also show that *Osmylus* eggshell, distinctive to other studied so far Neuroptera, exhibits not only AP but also DV polarity. The anterior pole of the eggshell is marked by the micropyle, while the ventral side is recognizable by the asymmetric position of the micropyle and micropylar canal openings.

Among insects, the mechanisms that govern the polarization of the oocyte and future embryo have been known from extensive studies in *Drosophila melanogaster*. It has been shown that in *Drosophila*, the AP and DV axes formation is very complex, and the key event implicated in this process is GV migration. In *Drosophila*, the AP and DV egg polarity corresponds to the polarity of the future embryo. Moreover, the egg axes show clear reflection in the complex structure of the eggshell (Gonzalez-Reyes and St Johnston 1994; González-Reyes et al. 1995; Roth 2003). The latter is equipped with several specialized structures such as the micropyle at the anterior pole, an aeropyle at the posterior pole, and respiratory appendages at the dorsal side (Wakimoto et al. 1986; Spradling 1993) whose position marks AP and DV axes. It has been demonstrated that in *Drosophila*, the establishment of axial polarity is realized through asymmetric localization of mRNA coding for TGFα-like epidermal growth factor Gurken (Grk) that is closely associated with the position of the germinal vesicle as it migrates to antero-dorsal region of the oocyte (Deng and Bownes 1998; for reviews, see e.g., Johnstone and Lasko 2001; Kugler and Lasko 2009). Gurken activity induces overlying follicular cells to form antero-dorsal chorion structures (i.e., respiratory appendages) and fixes the proper position of the micropyle. Genes encoding TGFα-like proteins, similar to two paralogs of Gurken: spitz and keren, were found in a few non-dipteran insects (Lynch et al. 2010). Whether the same signaling works in neuropteran insects still remains obscure, since molecular data concerning this issue is lacking.

Comparative morphological observations of follicular cell morphogenesis have been carried out in several brachycerans (higher Diptera) (Kubrakiewicz et al. 2003; Jaglarz et al. 2008, 2010) and nematocerans (lower Diptera) (Mazurkiewicz and Kubrakiewicz 2005, 2008; Mazurkiewicz-Kania et al. 2012). However, results of these studies do not suggest an influence of GV position on the follicular cell diversification. On a basis of morphological observations, the inductive role of GV on follicular cell morphogenesis has been postulated in some heteropterans (Ogorzałek 1987). It has been observed, that in water bugs, the position of GV is first of all diversified and is second, species specific. It has been found, that in different bug species, the oocyte nucleus eventually occupies different ooplasm portions: equatorial, subapical, or posterio-lateral. Moreover, the behavior of follicular cells in relation to GV neighborhood is diverse. For example, the follicular cells were found either to decrease in height, undergo hypertrophic growth, or form an epithelial pocket. In *Osmylus*, however, we did not observe any significant alterations of follicular morphology in the close vicinity to the GV. Although the influence of a dorsally positioned GV is not apparent, such a possibility cannot be excluded and requires further more detailed studies.

As it was indicated above, the position of the insects’ oocyte nucleus may be different and appears as a species- or taxon-specific character. It has been elegantly evidenced, that in the fruit fly, the GV takes a long-distance tour from a central location via the posterior pole to the antero-dorsal region of the oocyte. To our knowledge, the position of oocyte nucleus in neuropterans has not been previously studied. In this paper, we show that in *Osmylus*, during consecutive stages of oogenesis, the GV migrates from the central to the dorsal side of the oocyte and maintains its subcortical position halfway between anterior and posterior poles of developing egg. Similar positioning of the GV to that described in *Osmylus* has been previously reported in e.g., *Tribolium castaneum* (Coleoptera: Polyphaga *eng*. polyphagan beetles), *Gryllus bimaculatus* (Orthoptera *eng*. orthopterans) (Lynch et al. 2010), and *Notonecta glauca* (Heteroptera *eng*. true bugs) (Ogorzałek 1987). It should be also highlighted that asymmetric positioning of the oocyte nucleus correlating with the dorsal side of the egg has been considered as an ancestral insect character (Sander 1976). The results of our studies also show that asymmetric position of GV in *Osmylus* affects both the oocyte and the embryo DV polarization. In some insects, however, e.g., *Drosophila*, GV migration is crucial not only for dorsal patterning of the egg and future embryo, but also for DV polarity of the eggshell (Roth 2003). Whether the asymmetric position of the GV also influences DV polarity of the eggshell remains unclear. As we demonstrate, DV polarity of the eggshell in *Osmylus* results from asymmetric construction and position of the micropyle produced by the cells of anterior fold. Since these cells diversify in a long distance from GV, it seems unlikely that they are affected by any hypothetical factors emitted from the GV. Taken together, the inductive function of GV on the follicular cell diversification should be considered unclear at present. If one assumes that the GV does not induce alterations in specific follicular cell subpopulations, another possible explanation appears to be that of the follicular cell autonomy. The idea that the follicular cell developmental program is partly independent from germline-soma cell interactions, and thus also from the position of the GV, has been confirmed by experiments with *Drosophila* mutants (Gutzeit and Strauss 1989), as well as in comparative studies of follicular cell development in lower dipterans (Mazurkiewicz-Kania et al. 2012). It is tempting to assume that in *Osmylus*, like in other neuropterans, the final activity of follicular cells manifested by the micropyle production might be independent of GV influence.

One of the possible factors that might influence modifications in the eggshell structure is environmental requirements. It has been clearly demonstrated that the sculpture and structure of the eggshell is most often an environmental adaptation and usually depends on the site and manner of egg laying. In many neuropterans, eggs are laid in a vertical position on the stalks as, e.g., in *Chrysopa carnea* (Mazzini 1976) or into the sand as in antlions (Yasseri et al. 1996). In *O. fulvicephalus*, eggs are laid on an underside of a leaf and the position of the eggs is specific, because the eggs are stuck to the leaf by their dorsal side. Thus, the most probable explanation of asymmetric structure of the eggshell in *O. fulvicephalus* seems to be an adaptation to specific environmental conditions.
